# Local Anæthesia in General Medicine and Surgery

**Published:** 1886-03

**Authors:** 


**Local Anæsthesia in General Medicine and Surgery.** By J. LEONARD CORNING, M. D., author of Brain Rest, and formerly Resident Physician to the Hudson River State Hospital for the Insane, etc., etc. New York: D. Appleton & Co. 1886.

This is a little work in which is recorded the practical application of the author's recent discoveries. These discoveries have gained the author a well-deserved reputation among the profession. It gives many useful points in the alleviation of pain. The following subjects are treated of in the work: Cerebral anæsthesia; coca; cocaine; on the prolongation of the anæsthetic effects of the hydrochlorate of cocaine, when cutaneously and subcutaneously injected; minute directions concerning the practical execution of the author's method of local anæsthetization; incarceration of the anæsthetic by means of rings and hæmostatic clamps; local anæsthesia in bone surgery; on the prolonged duration of the anæsthesia after removal of the tourniquet; explanation of the phenomenon; operations upon soft tissues; the influence of cocaine upon healing of wounds; spinal anæsthesia and local medication of the cord. From this it will be seen that the book is of inestimable worth to the physician. It is well printed and nicely bound, making in all a publication of which Messrs. Appleton & Co. can be proud.

